# The effectiveness of using a WeChat account to improve exclusive breastfeeding in Huzhu County Qinghai Province, China: protocol for a randomized control trial

**DOI:** 10.1186/s12889-019-7676-2

**Published:** 2019-12-02

**Authors:** Qiong Wu, Yiwen Huang, Michelle Helena van Velthoven, Wei Wang, Suying Chang, Yanfeng Zhang

**Affiliations:** 1 Department of Integrated Early Childhood Development, Capital Institute of Pediatrics, No. 2 Yabao Road, Chaoyang District, Beijing, 100020 People’s Republic of China; 2 Department of Paediatrics, Level 2, Children’s Hospital, John Radcliffe, University of Oxford, Headington, Oxford, OX3 9DU UK; 3 Health and Nutrition, Water, Environment and Sanitation Section, UNICEF China, 12, Sanlitun Lu, Beijing, 100600 People’s Republic of China

**Keywords:** Breastfeeding, Exclusive breastfeeding, Mhealth, WeChat, Randomized controlled trial

## Abstract

**Background:**

An exclusive breastfeeding rate in the first 6 months of life of at least 50% is one of the six World Health Organization global nutrition targets for 2025. However, the exclusive breastfeeding rate in China is quite low and decreasing which makes it urgent to explore effective ways to reverse the current downward trend. As mobile technologies have rapidly developed, mobile communication apps such as WeChat (one of the largest social networking platforms in China) are well accepted and have the potential to improve health behaviors in a convenient way. The current paper describes the study protocol of a WeChat intervention that aims to promote breastfeeding in rural areas in China.

**Methods:**

The study is designed as a randomized controlled trial in rural Qinghai Province, China. Women who are 14–36 weeks pregnant will be randomized to routine antenatal and postnatal care, or routine care plus the WeChat breastfeeding education. pregnant women with a severe disease and complications of pregnancy or HIV-1 will be excluded. Breastfeeding knowledge and promotion information will be delivered to the intervention group through a WeChat official account from 3 months pregnancy to 6 months postpartum. The outcome assessments are conducted at baseline through face-to-face interviews, and at one week, 1 month, 3 months and 6 months postpartum by telephone interviews. The primary outcome is difference in the exclusive breastfeeding rate at 1 month, 3 months, and 6 months postpartum between the intervention group and the control group. Secondary outcomes include the overall duration of any and exclusive breastfeeding across the first 6 months postpartum; mothers’ knowledge of breastfeeding; the proportion of early initiation of breastfeeding; and the timing of the introduction of solid food to infants. Intention-to-treat-analysis will be used. Survival analysis will be used to compare the overall duration of any and exclusive breastfeeding between groups.

**Discussion:**

This study is the first effort to promote exclusive breastfeeding through WeChat in China. Our results will provide scientific evidence for the effect of health education through WeChat on breastfeeding. Thereby this may offer a comprehensive intervention to promote exclusive breastfeeding in China and other settings.

**Trial registration:**

Chinese Clinical Trial Registry –ChiCTR1800017364. Registered 26 July 2018. http://www.chictr.org.cn/showproj.aspx?proj=29325

## Background

Increasing the exclusive breastfeeding rate in the first 6 months of life to at least 50% is one of the 6 WHO global nutrition targets for 2025 [1]. The benefits of breastfeeding for both infants and mothers have been well recognized [2]. Exclusive breastfeeding provides infants with the best start to life by ensuring adequate nutrition and protection from infection, as well as fostering early growth and development. Therefore, the World Health Organization (WHO) and United Nations Children’s Fund (UNICEF) jointly recommend that infants should be exclusively breastfed for six months after birth, and be continuously breastfed to 2 years or beyond [3].

Over the past two decades, China has adopted the WHO’s feeding recommendations and implemented programs to promote breastfeeding and complementary feeding, such as the infant and young child feeding (IYCF) guidelines and the integrated management of childhood illness (IMCI) guidelines [4–6]. Moreover, China initiated a national Basic Public Health Service program in 2009 to provide universal basic public health services for all residents [7], for which maternal and child health care workers are required to provide face-to-face counseling to women during antenatal care visits, hospital delivery, newborn home, postnatal care and child health care visits [7]. Nevertheless, exclusive breastfeeding under six months in China is still suboptimal; the latest national Chinese data from 2013 showed that the exclusive breastfeeding rate under six months was only 18.6% [8], which was even lower than the rate in 2008 (27.6%) [9]. Research studies have also reported a range of low exclusive breastfeeding rates under six months in China: 28.7% in central and western China [10]; and 9.7% in a rural county in Northern China [11]. Recent data showed that the exclusive breastfeeding rate in infants younger than 6 months was 41% globally [12] and 37% in low-income or middle-income countries [2]. As the exclusive breastfeeding rate in China was even lower than the global average level, much effort is needed to promote breastfeeding, especially exclusive breastfeeding.

Breastfeeding practices are influenced by a wide range of socio-economic, cultural, and individual factors in China, including maternal age, illness, delivery patterns, premature delivery, lactation factors, food intake before breastfeeding, infant’s sucking ability, the mother’s milk supply, the intention to breastfeed, understanding of breastfeeding benefits, breastfeeding experience, returning to work, and family support [13–16]. The most successful interventions to promote exclusive breastfeeding contain education and support, which are the two main approaches to breastfeeding promotion [17]. Successful breastfeeding depends on antenatal and postnatal breastfeeding education and on the support provided by healthcare professionals [14]. Improved breastfeeding knowledge and attitudes toward breastfeeding are related to more positive breastfeeding outcomes. However, our previous data from a rural county in northern China suggested that only 37.3% mothers knew the accurate duration of exclusive breastfeeding, and fewer than 30% of mothers ever received feeding information during pregnancy or after delivery [12]. Moreover, around 80% of mothers’ sources of information were family members, neighbors, friends and popular media (newspaper, magazine, book, radio and television), rather than health facilities [12]. As health workers in health facilities only have limited time and resources to provide breastfeeding education, there is a great need to explore other approaches to deliver breastfeeding education and support to mothers.

Smartphones have become new channels and tools for information acquisition and exchange, and been enthusiastically adopted by the general public. Among instant-messaging apps for smartphones, WeChat (the Chinese name is *Weixin*) is regarded as one of the largest social networking platforms in China. WeChat is a free all-in-one communications app widely used for sending text and voice messages, video calls, sharing photos and ‘moments’ (updates on someone’s daily life), and playing games [18]. Since first launched in January 2011, there are more than one billion registered WeChat users throughout the world by the end of 2018 [18]. In 2018, WeChat gradually attracted nearly 902 million daily active users [18]. Furthermore, there is a popular functional module of WeChat called ‘WeChat official accounts’, which can be requested for free and used to disseminate information. WeChat users can subscribe to official accounts and receive selected news or information. Currently, the number of registered WeChat Official Account has reached 20 million [19]. WeChat has been used as a communication tool and been shown to positively impacts on changing selected health behaviours. Using a WeChat account as part of treatment and management of asthma can help patients to learn about their disease and medications, as well as improve disease control and therapy outcomes [20]. A health education intervention via a WeChat account proved to be an effective, sustainable, feasible, and well-accepted strategy for improving malaria health literacy among Chinese expatriates in Niger [21]. A WeChat account used to carry out a weight loss intervention showed that the more actively male participants used WeChat, the more weight they lost [22]. To our knowledge, no previous study has evaluated the effects of WeChat education on breastfeeding promotion. Therefore, we aim to undertake a randomized control trial to evaluate the effectiveness of a WeChat education intervention in improving the exclusive breastfeeding rate and the duration of exclusive breastfeeding in rural areas of China.

## Methods

### Design

**Fig. 1:**
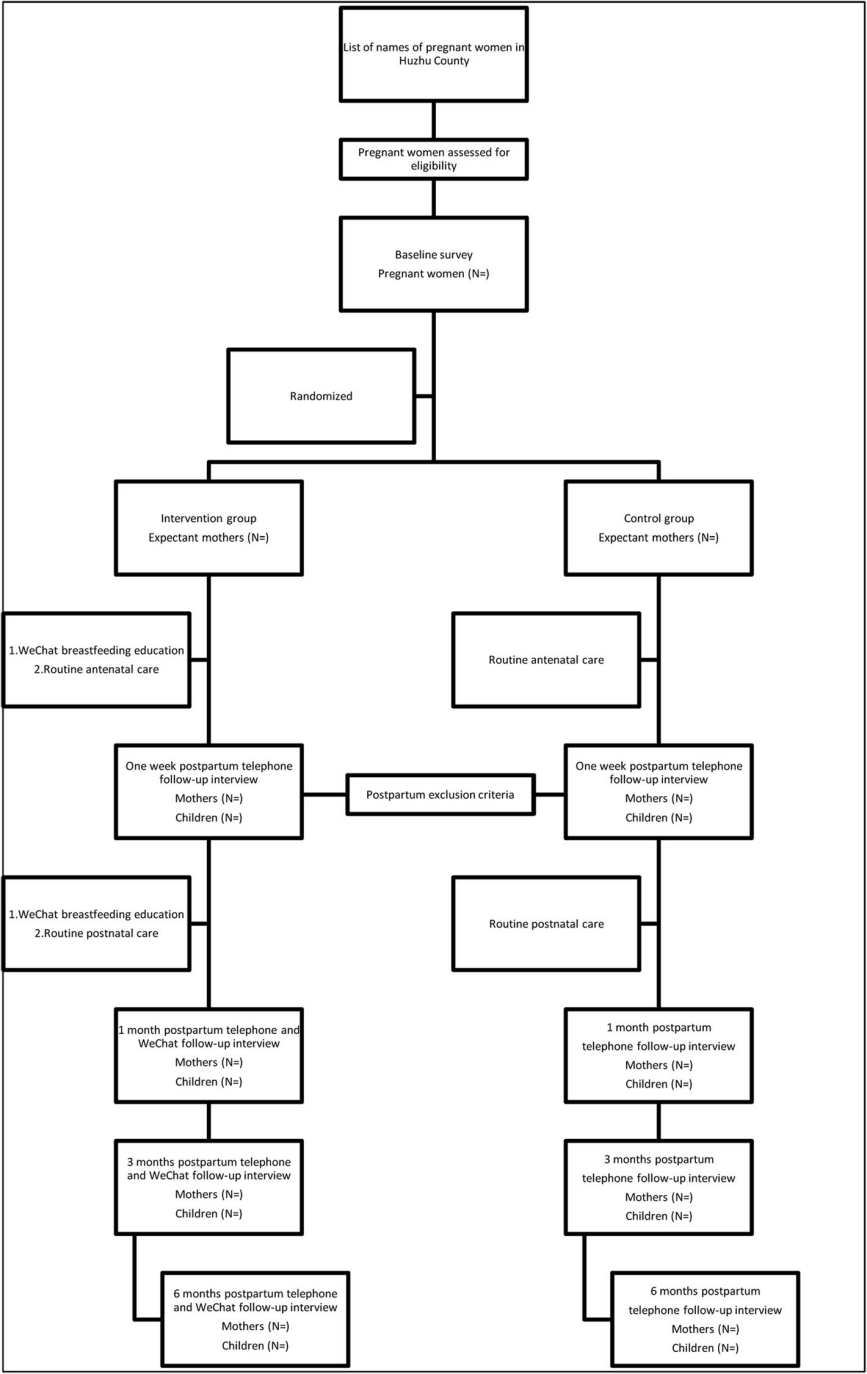
Study protocol flowchart of breastfeeding health education via an official WeChat account

This randomized controlled trial (RCT) will evaluate the effectiveness of using the official WeChat account called “Huzhu County Maternal and Child Health Family Planning Service Centre” in improving exclusive breastfeeding of children aged 0–6 months in rural Qinghai Province, China. Women pregnant 14–36 weeks will be randomized to routine antenatal and postnatal care or routine care plus the WeChat breastfeeding education (Fig. 1). Breastfeeding knowledge and promotion information will be delivered to the intervention group through the WeChat official account to women from the third month of their pregnancy until six months postpartum. The sampling unit is individual pregnant woman and we will enroll 200 pregnant women for both intervention and control group, respectively.

### Study area

This trial will be conducted in Huzhu County, Qinghai Province, China. Qinghai province lies in northwest China, with a surface area of 720,000 km^2^. By the end of 2017, the total population was 5,838,000 of which the rural population accounted for 46.9%. Qinghai Province has 34 counties and 439 townships. Huzhu County lies in the northeast of Qinghai province, with a surface area of 3423.9 km^2^. The county has a total population of 401,540 of which the rural population accounts for 76.0% and more than 80% people were Han Chinese. People in Huzhu County mainly make a living by farming. The annual per capita income of rural residents was Ұ9810 (US$1414.91) in 2017 [23]. In addition, there were a total of 91,321 women at reproductive ages and 4325 pregnant women in 2017.

Huzhu County has 19 townships, with 294 villages, of which we will select 13 townships as our study areas. Six townships will be excluded because whilst planning this study, 4 townships had already been selected by another maternal and child health project and another 2 townships are remote and only have a small number of pregnant women.

### Participants

We will enroll women who are 14–36 weeks pregnant from 13 townships in Huzhu County as our participants. Eligibility inclusion criteria will be: 1) first-time mothers aged 18 years old or above; 2) 14–36 weeks pregnant and conceiving a singleton fetus; 3) no known illness that limits breastfeeding after childbirth; 4) no long-term plan to go out of the county in the following year; 5) no plan to change their mobile phone number in the following year; 6) can read and communicate in Mandarin, use WeChat through smartphones, and have access to the internet; 7) informed consent and willing to cooperate actively. Participants with the following characteristics will be excluded: 1) pregnant women with a severe disease and complications of pregnancy, such as cerebrovascular diseases, pneumonia and threatened abortion; 2) pregnant women with HIV. Postpartum exclusion criteria will be: 1) mothers developing health complications after birth, such as acute uterine inversion, post partum hemorrhage or post partum depression; 2) infants who are admitted to the neonatal intensive care unit, have a 1-min Apgar score < 6, 5-min Apgar score < 6, low birth weight (< 2500 g), or were born prematurely (< 37 weeks of gestation); 3) infants who are diagnosed with a cleft palate or other congenital abnormality that will prohibit breastfeeding; 4) mothers and infants who move out or do not live in Huzhu County after birth.

### Sample size calculation

We will calculate the sample size required for this study based on previously collected data in Datong (another county in Qinghai), in which exclusive breastfeeding for 0–6 months children was only 29.2% (unpublished data in 2017). We expect to achieve a 20% increase in the exclusive breastfeeding rate by using the WeChat intervention. With a power of 80 and 5% significance level, we calculate that a sample size of 93 pregnant women respectively for intervention and control group will be sufficient for all the primary and secondary outcomes. To compensate for possible refusal and loss to follow-up, we will enroll 200 pregnant women for intervention and control group, respectively. To achieve this sample size, all eligible pregnant women in the 13 townships will be selected as participants over an estimated period of 15 days.

### Recruitment and randomization

Before the baseline survey, each township hospital will firstly provide a list of names of all women who are 14–36 weeks pregnant of their own township, which also includes information on gestational age, gravidity and parity. In addition, we will ask the township hospitals to highlight on the list pregnant women who are not at home all the year, do not receive antenatal care in township hospitals, or are unable to use a smartphone. Based on the inclusion and exclusion criteria, they will be asked to come to their own township hospitals. Those who meet the eligibility criteria will be given full information about the study. After signing the written informed consent and completing a basic demographic questionnaire, all the eligible participants will be randomized to either the WeChat intervention group or the control group at a 1:1 ratio.

An independent statistician who will not participate in recruitment or data collection will generate 400 random numbers using Microsoft Excel. She will use the median of the random numbers as a threshold. Random numbers lower or equal to the median will be allocated to the intervention group and random numbers higher than the median will be allocated to the control group. The random numbers and the allocated group will be printed on small cards, and kept in opaque, sealed envelopes. At the back of the random number card, the two-dimensional codes of the WeChat official account “Huzhu County Maternal and Child Health Family Planning Service Centre” will be printed. In addition, the two-dimensional code of “Assistant 1” will also be printed at the back of the random number card for intervention group and “Assistant 2” for control group. “Assistant 1” and “Assistant 2” will be used by study team members for future follow-up.

After an eligible pregnant woman agrees to participate and has signed the written consent form, a researcher will give the next envelope in sequence to her, and she will open the envelope to check the random number to determine the assigned group. All the pregnant women in both intervention group and control group will be asked to follow the WeChat account “Huzhu County Maternal and Child Health Family Planning Service Centre” on their smartphone through scanning the two-dimensional codes at the back of random number card. If the pregnant woman is assigned to the intervention group, the researcher will help her to subscribe to and register with the “ke Xue Wei Yang (Optimal feeding)” module in the official account, and make a brief introduction of the module. If the pregnant woman is assigned to the control group, the researcher will inform her about the study procedures, including the follow-up interviews. Both the intervention group and control group need to subscribe to the “Assistant” WeChat account for future follow-up. As pregnant women in the control group cannot register with the “ke Xue Wei Yang (Optimal feeding)” module, they cannot access to the information in the module, which avoids between group contaminations through direct sharing of messages sent via WeChat.

### Intervention

A WeChat official account will be used to deliver health education messages related to breastfeeding. The intervention is based on the existing WeChat official account “Huzhu County Maternal and Child Health Family Planning Service Centre” applied by Huzhu County Maternal and Child Health Family Planning Service Centre. The Huzhu County Maternal and Child Health Family Planning Service Centre and Capital Institute of Pediatrics jointly developed a special module “ke Xue Wei Yang (Optimal feeding)” for the WeChat official account with the technological support of a specialized information technology company (ZYZY (Beijing) Pioneer of Cultural Essence Co. Ltd.). All the participants in the intervention group will be asked to register with the module after completing the baseline survey. Before becoming members of the module, they will have to read the introduction of this study. They will be asked to register and provide information regarding their name, phone number, gestational age, expected date of delivery, village and county where they live. After they will be provided and can log in with a WeChat identification and password.

**Fig. 2:**
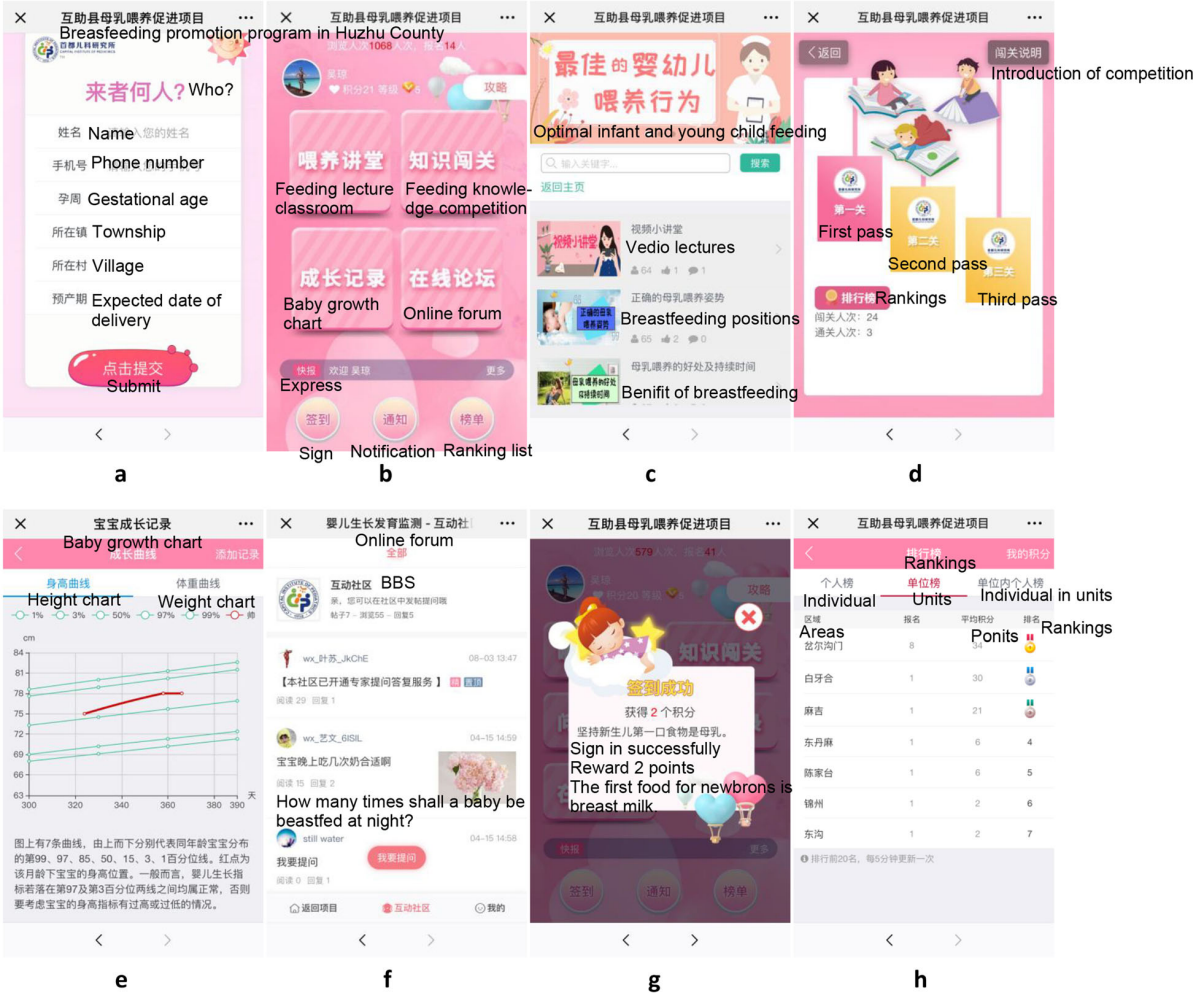
The interface of the WeChat interventions. (**a**) Login interface, (**b**) Main interface, (**c**) Feeding lecture classroom, (**d**) Feeding competition, (**e**) Baby growth chart, (**f**) Online forum, (**g**) Rewards, (**h**) Rankings

The official WeChat account includes the following four components: feeding lecture classroom, feeding knowledge competition, baby growth chart and online forum. Each component is described in detail (Fig. 2).

The ***feeding lecture classroom*** is designed to provide key breastfeeding knowledge and relevant infant feeding advice. Participants can read new messages and review the message history of all content published in the official account. The education messages will be shown in the form of text, videos, and pictures.

All breastfeeding health education messages are developed based on the WHO breastfeeding recommendations, guidelines, and published literature [3–6, 13–16, 24–27]. There are four phases in the development of health education messages: 1) a breastfeeding information database was first developed according to the recommendations, guidelines, and published literatures, which will include all the related breastfeeding themes and the key themes of this database are summarized in Table 1; 2) a research group meeting was held to identify all the detailed messages that will be published in the feeding lecture classroom; 3) each message will be compiled by one research team member who will develop appropriate multimedia content using videos, pictures, or records to effectively convey the meaning of each message and each message will be revised with expert opinion and subsequently approved by all research team members; 4) another research team member will be responsible for publishing all the messages in the feeding lecture classroom in the official account “Huzhu County Maternal and Child Health Family Planning Service Centre” before baseline. All the pregnant women and mothers in the intervention group can log into the feeding lecture classroom to read and learn all those breastfeeding messages.

**Table 1:** Key themes of breastfeeding promotion education

Category	Key themes
Core messages	➢ Importance and benefits of exclusive breastfeeding ➢ Early initiation breastfeeding ➢ Cues to start feeding and the signs of infant satisfaction ➢ Breastfeeding on demand ➢ Frequency of breastfeeding ➢ Breastfeeding positions and latching-on ➢ Benefits of breastfeeding ➢ Weaning time ➢ Normal milk supply ➢ Rooming-in
Breastfeeding problems encountered for both mother and child	➢ Return to work and breastfeeding ➢ Risks of formula supplements ➢ Specific guidance for women who had cesarean section delivery ➢ Determining adequate intake ➢ Common misunderstandings about breast feeding
Preparation	➢ Preparation for breastfeeding ➢ Preparation for starting solid foods at 6 months

In addition, we suppose that there are three key stages for mothers, late pregnancy (37 weeks or above), the first month postpartum and 4 months postpartum, during which breastfeeding information needs to be strengthened. Three sets of tailored messages will be sent to all pregnant women and mothers at these stages via their WeChat on Monday, Wednesday and Friday every week. Specifically, for the late pregnancy stage, information on getting ready for breastfeeding and key breastfeeding recommendations will be sent to pregnant women whose gestational weeks are 37 weeks or above. For the first month postpartum stage, the key breastfeeding recommendations and breastfeeding problems encountered for both mother and child will be sent to new mothers. For the 4 months postpartum stage, information on starting complementary feeding at 6 months to avoid introducing complementary food too early or too late will be sent to mothers whose children were 4 months old or above.

The ***feeding knowledge competition*** component aims to test how well mothers know the breastfeeding knowledge. Participants need to participate once in the third and fourth week of each month.

The ***baby growth chart*** component is developed based on the WHO growth chart standard. Mothers can enter data on weight and height of their children whenever they want to monitor their children’s growth. Once the data are reported, a feedback message on whether their children’s weight and height are between the 3rd and 97th percentiles will be provided to mothers immediately.

The ***online forum*** component is designed to provide a feedback activity between mothers and experts. Mothers can ask breastfeeding related questions on the forum and three experts from the Capital Institute of Pediatrics will give feedback answers through the WeChat platform. Once the feedback is send, mothers will receive a WeChat message which reminds them to review the feedback answers.

Additionally, there will be an “Express” function which will send system notification alert, including updated feeding messages, competition and questionnaires.

A **rewards component** will also be developed to stimulate mothers to participate in our study. During the intervention, mothers can receive scores as long as they participate in WeChat activities. Different types of activities will result in different scores (Table 2). We will rank the scores mothers receive every month, and the top 10 WeChat active mothers will be given rewards, such as toys, picture books, or children towels.

**Table 2:** Scores received from activities

Activities	Scores (points)
Registration	20
Read and review the breastfeeding messages in the feeding lecture classroom	5 per message
Share learning experience	2 per message
Score the feeding messages	1 per message
Feeding knowledge competition	2 per time
Questionnaires	20 per time
Baby growth chart	2 per time

The “ke Xue Wei Yang (Optimal feeding)” module was pre-tested in Huzhu County in Aug 2018. Semi-structured interviews were conducted among 17 pregnant women in their second or third trimester and 16 mothers who had a child aged 0–6 months to learn their use experience of the platform to learn from their experience in using the platform. They generally accepted the platform, as they thought the information was easy to understand and useful. They also suggested that both videos and text&picture should be delivered to mothers, as some mothers like video, while some like text&pictures. The detailed description of the methods and results of the pre-test will be reported elsewhere (manuscript in preparation).

### Control group

Mothers in the control group will only receive the routine antenatal and postnatal health care services (as will the intervention group). Participating infants in both groups will receive routine physical checkups (1, 3, 6, 9 and 12 months) in their own village clinics during their first year [7]. Although participants in the control group will also follow the “Huzhu County Maternal and Child Health Family Planning Service Centre” WeChat official account, they cannot log in the “ke Xue Wei Yang (Optimal feeding)” module and get feeding information from the module.

### Outcome measurement

The primary outcome in this study will be the exclusive breastfeeding rate at month 1, month 3, and month 6 postpartum in both the intervention group and the control group. Secondary outcomes for both intervention group and control group will be as following: 1) the overall duration of any and exclusive breastfeeding across the first 6 months postpartum; 2) mothers’ knowledge on breastfeeding practices; 3) the proportion of early initiation of breastfeeding; 4) the timing of the introduction of solid food to infants.

Exclusive breastfeeding will be defined according to the WHO definition which says that the infant receives breast milk as the only source of food; no other liquids or solids are allowed with the exception of vitamin and mineral supplements or medication [28]. The duration of exclusive breastfeeding is defined as the time in weeks until mothers introduce food or drink except for breast milk (we will ask mothers in the follow-up survey “When did your child first eat or drink other food or liquid except for breast milk, including drinking water?” ).

Any breastfeeding will be defined as the infant receiving any breast milk along with other food or liquids, including formula. The duration of any breastfeeding is defined as the time in weeks until a mother stops breastfeeding completely (we will ask mothers in the follow-up survey “When did you completely stop breastfeeding to your child?”).

Early initiation of breastfeeding will be defined based on the WHO guideline as the infant being put to the mother’s breast within one hour of birth [28].

### Data collection

Before the intervention, a baseline survey will be carried out via face-to-face interviews to collect data of all participants on demographic characteristics information, antenatal care, as well as breastfeeding knowledge and channels through which they receive information on breasfeeding.

Pregnant women will be asked to come to township hospitals. Those who meet the eligibility criteria will be given full information about the study. After signing the written informed consent, they will be interviewed to collect baseline data. Smartphones will be used to collect data. Two supervisors and 10 interviewers will conduct the baseline survey. Experienced staffs from the Capital Institute of Pediatrics will be the study supervisors, and medical students form Huzhu County Vocational Technical School will be recruited as interviewers. We will conduct one day training for the interviewers, which will include questionnaire explanation, smartphone usage for collecting data, and role play.

Research assistants will be recruited from the Qinghai Institute of Health Sciences to conduct follow-up telephone interviewers at week 1, month 1, month 3 and 6 months postpartum for both the intervention and control group. Follow-up data will be collected on maternal breastfeeding knowledge, practices, reasons for weaning and information channels by smartphones (Additional file 1).

### Data management and analysis

One research staff member blinded to the treatment allocation will be in charge of database maintenance and management. Logic verification statements will be written in data acquisition software for the smartphone. Baseline data and follow-up data will be saved first on the smartphone, and then wirelessly uploaded into an Excel database via an internet server [29].

Statistical analyses will be performed by the research staff using SAS 9.2 software (SAS Institute, Cary, NC). Continuous variables will be presented as mean and deviation, while categorical variables as numbers and proportions. The homogeneity in baseline characteristics between the intervention group and control group will be estimated using t tests for continuous variables andχ^2^ test for categorical variables. In addition, theχ^2^ test will also be used to compare the differences of exclusive breastfeeding rate between two group at one week, 1 month, 3 months, and 6 months postpartum. Survival analysis will be used to compare the overall duration of any and exclusive breastfeeding between the groups. We will construct Kaplan-Meier survival curves to estimate the median exclusive breastfeeding times and use the log-rank test to compare the differences. We will also perform Cox proportional hazards regression to estimate hazard ratios for stopping exclusive breastfeeding or any breastfeeding.

The principle of intention-to-treat-analysis will be used. This means that participants who drop out or are lost to follow-up during the postpartum period will be considered to have stopped breastfeeding at the point of the last follow-up contact and all randomized participants will be included in the analysis. We will further calculate the odds ratios of any and exclusive breastfeeding at each follow-up time point using logistic regression. All statistical tests will be two-tailed and *P* < 0.05 will be considered significant.

## Discussion

This paper describes the study protocol for a randomized controlled trial of a WeChat breastfeeding promotion, which will aim to encourage mothers to exclusively breastfeed their children if they can in the first 6 months postpartum. To our knowledge, this will be the first randomized controlled trial of using WeChat to support mothers on breastfeeding in China.

In China, the exclusive breastfeeding rate is quite low. Moreover, national data showed it decreased by more than one percentage point annually between 2008 and 2013 [8]. It is urgent to explore effective ways to control this down trend, and research in this area is needed. Given the widespread use of WeChat and a large number of active users, WeChat can be an important health promotion tool in China. Therefore, our study will provide important insights in using a WeChat intervention to improve the exclusive breastfeeding rate in China.

Our study will examine the WeChat intervention’s effect on the exclusive breastfeeding rate and duration using a rigorous randomized controlled trial design, which will ensure balance among the confounding variables in each group and eliminate the bias caused by characteristics of the participants. We hypothesize that the intervention group will have a higher exclusive breastfeeding rate and longer exclusive breastfeeding duration than in the control group. Our WeChat intervention delivery is based on the local county WeChat official account, which makes the program sustainable as the county will be able to continue using the account.

However, our study also has several limitations. First, all participants will be recruited from one rural county in China, and we also exclude two remote townships, which limits generalizability. Second, there will be selection bias during the entire process, as enrolled participants are required to use the WeChat app. Therefore, we will strictly follow the inclusion criteria when recruiting participants and use methods to blind data collection during the implementation phase. Third, given the popular use of WeChat, there are lots of other WeChat official accounts on breastfeeding promotion, and we cannot guarantee that each participant in both groups will not receive knowledge regarding breastfeeding from other sources during the intervention period. However, our RCT design could limit such bias. In addition, as randomization units are individual pregnant women, contamination may exist between intervention and control groups within the same township.

In conclusion, this study will provide scientific evidence on the effectiveness of using WeChat to improve exclusive breastfeeding in rural China. The recruitment phase of this study is scheduled for May 2019. The breastfeeding intervention will be conducted for at least six months for each mother. Follow-ups will be conducted over 6 months and we will report the intervention effects when the follow-ups conclude.

## Supplementary information

**Additional file 1.** Follow-up questionnaires at week 1, month 1, month 3 and month 6 postpartum.

## Data Availability

The datasets generated and/or analyzed in the current study are available from the corresponding authors upon reasonable request.

## Abbreviations

IMCI: Integrated management of childhood illness
IYCF: Infant and young child feeding
RCT: Randomized controlled trial
UNICEF: The United Nations Children’s Fund
WHO: The World Health Organization
