# Supplementary material for: The effectiveness of using a WeChat account to improve exclusive breastfeeding in Huzhu County Qinghai Province, China: protocol for a randomized control trial
Source: BMC Public Health. 2019 Dec 2;19:1603. doi: 10.1186/s12889-019-7676-2 (PMC6889429; doi:10.1186/s12889-019-7676-2)
Supplement: Supplementary file 1 — Additional file 1. Follow-up questionnaires at week 1, month 1, month 3 and month 6 postpartum. [file 12889_2019_7676_MOESM1_ESM.docx]

**Follow-up questionnaire**

| **ID.1a** | Township___________ |
| --- | --- |
| **ID.1b** | Village___________ |
| **ID.2** | Mother’s name___________ |
| **ID.3** | Child name_________________ |
| **ID.4** | Child sex:1.Male 2.Female |
| **ID.5** | Birth of date：(year/month/day)________/___/___ |
| **ID.6** | Interviewee：___________ |
| **ID.7** | Survey date：(year/month/day) ________/___/___ |

| **Delivery** | |
| --- | --- |
| **DN.1** | Where did you give birth to your child？ |
|  | 1. At home  2. Huzhu People’s hospital  3. Huzhu maternal and child health hospital  4. Huzhu traditional Chinese medicine [hospital](http://cn.bing.com/dict/search?q=hospital&FORM=BDVSP6&mkt=zh-cn)[,](http://cn.bing.com/dict/search?q=%2C&FORM=BDVSP6&mkt=zh-cn)  5. township hospitals  6. Private hospitals in Huzhu County  7. Hospitals out of Huzhu County |
| **DN.2** | Did you give birth by cesarean section? 1.Yes 2.No |
| **DN.3** | What’s your gestational age when you gave birth？ __ __weeks（88=Do not know） |
| **Early initiation of breastfeeding** | |
| **EB.1** | How long after birth was your child first put to the breast? |
|  | 1. Within first hour after delivery **——>*Ship to BN.4***  2. After first hour, ______hours（88=Do not know）  8. Don’ know**——> *Ship to BN.4*** |
| **EB.2** | Why didn’t you put your child to your breast within the first hour after delivery? |
|  | 1. Child should be feed water or sugar water first after delivery 2. Suggestions from relatives 3. Suggestions from doctors 4. cesarean section 5. Don't know early initial 6. More than one hour after delivery when saw my child 7. I didn’t have breast milk 8. Others, specify________________   88. Don't know |
| **EB.4** | Did you feed your child colostrum? |
|  | 1. Yes**——>skip to BN.5**  2. No |
| **EB.4.a** | Why didn’t you feed your child colostrum? |
|  | 1. 1. Child should be feed water or sugar water first after delivery 2. 2. Suggestions from relatives 3. 3. Hospital provide infant formula 4. 4. Child and mother are not in the same room 5. 5. Others, specify________________ |
| **EB.5** | Did you feed your child other drink or food before feeding breast milk?   1. Yes 2. No**——>ship to BN6** |
| **EB.5a** | What did you feed your child before feeding breast milk?   1. Plain water 2. Sugar water 3. Infant formula 4. Milk 5. Others, specify________________ |
| **EB.6** | Did you breastfeed your child on demand or feed on time?  1. On demand  2. On time |

| **Breastfeeding and Nutrition** | |
| --- | --- |
| **BN.1** | Can you tell me until what age a baby should only receive breastmilk, i.e. no other food, water or fourmal) |
|  | __ __months [0=less than one month, 88=Don’t know] |
| **BN.2** | Can you tell me until what age a baby should start receiving foods such as mashed or solid foods? |
|  | __ __ months [0=less than one month, 88=Don’t know] |
| **BN.3** | Can you tell me until what age a baby should be breastfed? |
|  | __ __months [0=less than one month, 88=Don’t know, 90=when the milk dries out or when the child no longer wants the breast] |
| **BN.4** | Call you tell the conception of exclusive breastfeeding in your opinion? |
|  | 1. Only feed breast milk，no other food, water or drink 2. Except for breast milk, child can also be fed water 3. Except for breast milk, child can also be fed water or drinks 4. Except for breast milk, child can also be fed water, drinks or other foods such as thin porridge. 5. Don’t know |
| **BN.5** | Do you think how longer after birth should a baby first put to the breast? |
|  | After__ __hours  88.Don’t know |

| **BN.6** | Has your child ever been breastfed? |
| --- | --- |
|  | 1. Yes 2. No |
| **BN.7.a** | Did you feed your child eat or drink other food or liquid except for breast milk?   1. Yes 2. **No——>ship to BN7** |
| **BN.7.b** | When did your child first eat or drink other food or liquid except for breast milk, including drinking water?  __ __weeks [0=less than one week, 88=Don’t know] |
| **BN.7.d** | What other food or liquid except for breast milk did you first feed to your child? |
|  | 1. Plain water 2. Sugar water 3. Infant formula 4. Milk 5. Thin porridge 6. Drinks 7. Others, specify________________ |
| **BN.7** | Was your child breastfed yesterday during the day or at night？ |
|  | 1. Yes——>How many times did you breastfeed your child during the past 24 hours? __ __ *times [88=Don’t know]***——> *ship to BN.8***  2. No. my child did not eat breast milk.**——> *ship to BN.8*** |
|  | 1. No. My child has been stopped breastfeeding**——>** When did you completely stop breastfeeding to your child?？__ __ weeks [0=less than one week, 88=Don’t know] |
| **BN.7.c** | Why did you stop breastfeeding ？ |
|  | 1. I have no breast milk supply 2. My breast milk was no longer nutritious 3. My child grows up 4. I have to go back to work 5. I got illness at that time 6. Others, specify________________ _   8. Don’t know |

| **BN.8** | Next I would like to ask you about some liquids that your child may have had yesterday during the day or at night. Did your child have any (item from List)?: read the List of Liquids starting with ‘Plain water’. | |
| --- | --- | --- |
| 1. Plain water / Spring Mineral Water /Sugar water /tea | | 1.Yes 2.No 8.Don’t know |
| 2.Drinks/soups/rice soup | | 1.Yes 2.No 8.Don’t know |
| 3. Thin porridge | | 1.Yes 2.No 8.Don’t know |
| 4. Other liquids | | 1.Yes 2.No 8.Don’t know |
| 5. Infant formula/ milk such as tinned, powdered, or fresh animal milk/ yogurt? | | 1.Yes  2.No **——>skip to BN9**  8.Don’t know**——> skip to BN9** |
| **BN.8.5a** How many times yesterday during the day or at night did your child consume any infant formula/ milk such as tinned, powdered, or fresh animal milk/ yogurt? __ __ times | | |

| **BN.9** | Please recall everything that your child ate yesterday during the day or night, whether at home or outside the home.  Yesterday during the day or night, did your child drink/eat any (food group items below)? | |
| --- | --- | --- |
|  | 1. Porridge, bread, rice, noodles, or other foods made from grains | 1.Yes 2.No 8.Don’t know |
|  | 2. White potatoes, white yams, manioc, cassava, or any other foods made from roots | 1.Yes 2.No 8.Don’t know |
|  | 1. Any dark green leafy vegetables, or pumpkin, carrots, squash, or sweet potatoes that are yellow or orange inside | 1.Yes 2.No 8.Don’t know |
|  | 1. Any other fruits or vegetables | 1.Yes 2.No 8.Don’t know |
|  | 1. Fresh, such as beef, pork, lamb, goat, chicken, or duck   or dried fish, shellfish, or seafood, or liver. | 1.Yes 2.No 8.Don’t know |
|  | 1. Eggs | 1.Yes 2.No 8.Don’t know |
|  | 1. Any foods made from beans, peas, lentils, nuts, or seeds | 1.Yes 2.No 8.Don’t know |
|  | 1. Nuts | 1.Yes 2.No 8.Don’t know |
| **BN.10** | Has your child ever eaten solid, semi-solid or soft food other than liquids? |  |
|  | 1. Yes, when did he/she first eat? __ __week [0=less than one week] 2. No**——>转到BN.12** |  |
| **BN.11** | How many times did your child eat solid, semi-solid or soft food other than liquids yesterday during the day or at night? |  |
|  | ___ times *[If ≥7，fill”7”，8=Don’t know]* |  |

| **BN.12** | Did you receive advice or information on breastfeeding during pregnancy or after delivery? |
| --- | --- |
|  | 1. Yes  2. No**——>BN13**  8. Do not know **——>BN13** |

| **BN.12a** | Where did you receive advice or information on breastfeeding? Record all sources mentioned | |
| --- | --- | --- |
|  | 11 Relatives  12 Friends or neighbours | 1.Yes 2.No  1.Yes 2.No |
|  | 21County hospitals or above  22Township hospitals  23 Village clinics | 1.Yes 2.No  1.Yes 2.No  1.Yes 2.No |
|  | 31 Private hospitals  32 Private clinics | 1.Yes 2.No  1.Yes 2.No |
|  | 41 Internet  42 Newspaper/magazine  43 Broadcast /TV  44 WeChat | 1.Yes 2.No  1.Yes 2.No  1.Yes 2.No  1.Yes 2.No |
|  | 51.Books | 1.Yes 2.No |
|  | 61. Others, specify________________ _ | 1.Yes 2.No |
| **BN.12b** | Have you ever receive information on breastfeeding from “Huzhu County Maternal and Child Health Family Planning Service Centre” official WeChat account? | |
|  | 1.Yes 2.No | |

| **BN.13** | Did you receive advice or information on infant formula during pregnancy or after delivery? |
| --- | --- |
|  | 1. Yes  2. No**——>End**  8. Do not know **——>End** |

| **BN.13a** | Where did you receive advice or information on infant formula ? Record all sources mentioned | |
| --- | --- | --- |
|  | 11 Relatives  12 Friends or neiborge | 1.Yes 2.No  1.Yes 2.No |
|  | 21County hospitals or above  22Township hospitals  23 Village clinics | 1.Yes 2.No  1.Yes 2.No  1.Yes 2.No |
|  | 31 Private hospitals  32 Private clinics | 1.Yes 2.No  1.Yes 2.No |
|  | 41 Internet  42 Newspaper/magazine  43 Broadcast /TV  44 WeChat | 1.Yes 2.No  1.Yes 2.No  1.Yes 2.No  1.Yes 2.No |
|  | 51.Infant formula factories | 1.Yes 2.No |
|  | 61. Others, specify________________ _ | 1.Yes 2.No |
